# Poisoning by Sodium Salicylate

**Published:** 1879-09

**Authors:** 


### POISONING BY SODIUM SALICYLATE.

Dr. Feltz reports an interesting case, illustrating the toxic effects of sodium salicylate used continuously for several weeks, in quantity amounting to one and a half, and finally two drachms daily.

The total amount taken was about six and a half ounces. The toxic symptoms were principally, frequent vomiting, and repeated attacks of very painful headache, preceded by reddening of the neck, face and head. The pupils were much contracted and the symptoms continued for seventeen days after the last dose was taken, while the acid could be detected in the urine for sixteen days.

